# Bioprospecting of extremophilic perchlorate-reducing bacteria: report of promising *Bacillus* spp. isolated from sediments of the bay of Cartagena, Colombia

**DOI:** 10.1007/s10532-024-10079-0

**Published:** 2024-04-16

**Authors:** Rosa Acevedo-Barrios, Irina Tirado-Ballestas, Angela Bertel-Sevilla, Leonor Cervantes-Ceballos, Jorge L. Gallego, María Angélica Leal, David Tovar, Jesús Olivero-Verbel

**Affiliations:** 1 Environmental and Computational Chemistry Group, School of Pharmaceutical Sciences, University of Cartagena, Zaragocilla Campus, Cartagena, 130015 Colombia; 2 Grupo de Estudios Químicos y Biológicos, Facultad de Ciencias Básicas, Universidad Tecnológica de Bolívar, POB 130001, Cartagena de Indias D. T. y C, Colombia; 3 GENOMA Group, Health Sciences Department, Universidad del Sinú, Santillana Campus, Cartagena, 130015 Colombia; 4 Group of Functional Toxicology, School of Pharmaceutical Sciences, University of Cartagena, Zaragocilla Campus, Cartagena, 130015 Colombia; 5 Department of Engineering, University of Medellin, Medellín, 050026 Colombia; 6 Planetary Sciences and Astrobiology Research Group (GCPA), Universidad Nacional de Colombia and Corporación Científica Laguna, Bogotá, 111321 Colombia; 7 Biosphere and Cosmos Research Group (BIOC). Corporación Científica Laguna, Bogotá, 111163 Colombia

**Keywords:** Bioremediation, Halophytes, Marine sediment, RNA 16S

## Abstract

Three extremophile bacterial strains (BBCOL-009, BBCOL-014 and BBCOL-015), capable of degrading high concentrations of perchlorate at a range of pH (6.5 to 10.0), were isolated from Colombian Caribbean Coast sediments. Morphological features included Gram negative strain bacilli with sizes averaged of 1.75 × 0.95, 2.32 × 0.65 and 3.08 × 0.70 μm, respectively. The reported strains tolerate a wide range of pH (6.5 to 10.0); concentrations of NaCl (3.5 to 7.5% w/v) and KClO_4_^−^ (250 to 10000 mg/L), reduction of KClO_4_^−^ from 10 to 25%. LB broth with NaCl (3.5–30% w/v) and KClO4ˉ (250-10000 mg/L) were used in independent trials to evaluate susceptibility to salinity and perchlorate, respectively. Isolates increased their biomass at 7.5 % (w/v) NaCl with optimal development at 3.5 % NaCl. Subsequently, ClO_4_ˉ reduction was assessed using LB medium with 3.5% NaCl and 10000 mg/L ClO_4_ˉ. BBCOL-009, BBCOL-014 and BBCOL-015 achieved 10%, 17%, and 25% reduction of ClO_4_ˉ, respectively. The 16 S rRNA gene sequence grouped them as *Bacillus flexus* T6186-2, *Bacillus marisflavi* TF-11 (T), and *Bacillus vietnamensis* 15 − 1 (T) respectively, with < 97.5% homology. In addition, antimicrobial resistance to ertapenem, vancomycine, amoxicillin clavulanate, penicillin, and erythromycin was present in all the isolates, indicating their high adaptability to stressful environments. The isolated strains from marine sediments in Cartagena Bay, Colombia are suitable candidates to reduce perchlorate contamination in different environments. Although the primary focus of the study of perchlorate-reducing and resistant bacteria is in the ecological and agricultural realms, from an astrobiological perspective, perchlorate-resistant bacteria serve as models for astrobiological investigations.

## Introduction

The advancement of technology and industry has produced complex and toxic waste, leading to increased disposal into water, soil, and air, posing health and environmental risks (Gavrilescu et al. 2015). Conventional methods to tackle contamination are ineffective, expensive, and may exacerbate the issue. Hence, there’s a rising interest in biological remediation methods for their cost-effectiveness, high efficacy, and environmentally friendly (Liu et al. 2015; Azubuike et al. 2016).

Bioremediation utilizes microorganisms such as bacteria, archaea, fungi, and plants to absorb, transform, and degrade pollutants in soils, sediments, water, and air (Fenical 1993; Zolkefli et al. 2020; Belal et al. 2020). By immobilizing or altering the chemical structure of pollutants, bioremediation can lead to their partial degradation, mineralization, or transformation. Given the widespread contamination from human activities, pollutants are pervasive in various habitats, including extreme environments (Azubuike et al. 2016).

Microorganisms known as extremophiles have evolved specialized traits enabling them to thrive in harsh conditions, making them promising for biotechnological applications (Le Borgne et al. 2008). Marine microbial consortiums excel in diverse environmental variables like salinity, pH, and temperature. Bacteria from marine environments express genes for survival in saline conditions and have shown resilience in saturated matrices (Dalmaso et al. 2015). They also produce unique metabolites, enhancing their potential for bioremediation efforts amidst toxic pollutants (Bertel-Sevilla et al. 2020; Lee et al. 2021).

Some autochthonous extremophile strains generate biohydrogen as a substrate to treat organic waste to survive and use the macromolecules present in polluted marine environments as a carbon source (Oguntoyinbo 2007; Lee et al. 2009). In fact, halophytes strains are examples of extremophile microorganisms found in marine environments, with a remarkable ability to withstand harsh conditions and decontaminate the environment. Marine sediments are characterized by being the habitat of many native species and having the bioactivity of degrading xenobiotic substrates (Acevedo-Barrios et al. 2019, 2022), due to their resistance and efficient increase of biomass under extreme conditions, e.g., high salt concentrations (Cang et al. 2004; Van Ginkel et al. 2008; Srinivasan and Viraraghavan 2009). Hence, halophyte bacterial bioremediation will contribute to new alternatives for cleaning stressed environments.

The recent identification of perchlorate’s widespread occurrence in the environment, including its detection on Mars, the Earth’s moon, and in meteorites, as well as its involvement in regulating sulfidogenesis in oil reservoirs, has reignited interest in this unique ion and its microbiological implications (Carlström et al. 2016; Acevedo-Barrios and Olivero-Verbel 2021). Perchlorate, even at minute concentrations, poses significant health risks due to its toxic effects on the human thyroid gland (Shih et al. 2024). While current removal techniques like ion exchange and biological reduction show promise, their efficacy may be limited when applied individually (Shang et al. 2018; Xie et al. 2018; Fang and Naidu 2023). Integration of physico-chemical and biological processes appears essential for achieving comprehensive perchlorate decontamination (Shang et al. 2018; Xie et al. 2018). Extremophile-based bioremediation, particularly in marine environments, holds promise for perchlorate removal, as evidenced by studies isolating microorganisms capable of reducing perchlorate in diverse marine sediments (Acevedo-Barrios et al. 2023; Dong et al. 2022). For instance, in a study conducted by Acevedo-Barrios et al. (2019), marine sediment samples from different locations in the Colombian Caribbean were found to harbor various species of bacteria, such as members of the *Bacillus* genus, with a high potential for perchlorate reduction.

*Bacillus* spp. are Gram-positive bacteria widely recognized for their adaptability and resilience. They encompass over 222 species and exhibit characteristic features such as the production of spores, which enable them to endure adverse conditions (Garrity 2001; Miranda et al. 2008; Layton et al. 2011). These bacteria are commonly found in diverse environments, including deserts, marine ecosystems, and other harsh habitats, owing to their high biochemical activity and metabolic efficiency (Miranda et al. 2008; Elisashvili et al. 2019). *Bacillus* spp. have been isolated from various sources such as rice paddy soils (Beneduzi et al. 2008; Sriariyanun et al. 2016), pesticide-contaminated soils (Marín and Jaramillo 2015), perchlorate-contaminated soils (Acevedo-Barrios et al. 2016, 2019, 2022; Acevedo-Barrios and Olivero-Verbel 2021), hydrocarbons and their derivatives, liquid effluents from industries (Oyetibo et al. 2017; Masika et al. 2020), hypersaline mats, alkaline environments, i.e. food, sea water, or waste-water (Guadie et al. 2017; Durval et al. 2019; Mohapatra et al. 2019), and the intestinal tracks of insects and mammals (Hong et al. 2009). Furthermore, some *Bacillus* species have been shown to be capable of degrading some pollutants, including polycyclic aromatic hydrocarbons, long-chain alkanes (C10 to C32), and perchlorate (Feitkenhauer et al. 2003; Acevedo-Barrios et al. 2019). This versatility suggests that *Bacillus* spp. could serve as a basis for biotechnological methods aimed at mitigating environmental pollution.

The Bay of Cartagena, Colombia, is a tropical estuary in the Caribbean with an important biodiversity, but also historically affected by various sources of contamination (Romero-Murillo et al. 2023). This makes it an interesting area for the prospection of microorganisms for biotechnological applications. Our hypothesis lies in the reduction of perchlorate capacity in varied environmental conditions, of *Bacillus* genus strains isolated from the Colombian Caribbean.

Therefore, the objectives were to isolate and characterize halotolerant bacteria and evaluate their susceptibility and ability to reduce perchlorate in several pH and salinity conditions. This is the first study carried out in Bahía Cartagena, Colombian Caribbean; and it is part of our efforts to contribute to the knowledge of Colombian biodiversity with biotechnological potential. These bacteria of the genus *Bacillus* present suitable properties for possible biotechnological applications and constitute the basis for expanding our knowledge of salt-tolerant bacteria that can reduce perchlorate.

## Materials and methods

### Study area and sample collection

**Fig. 1:**
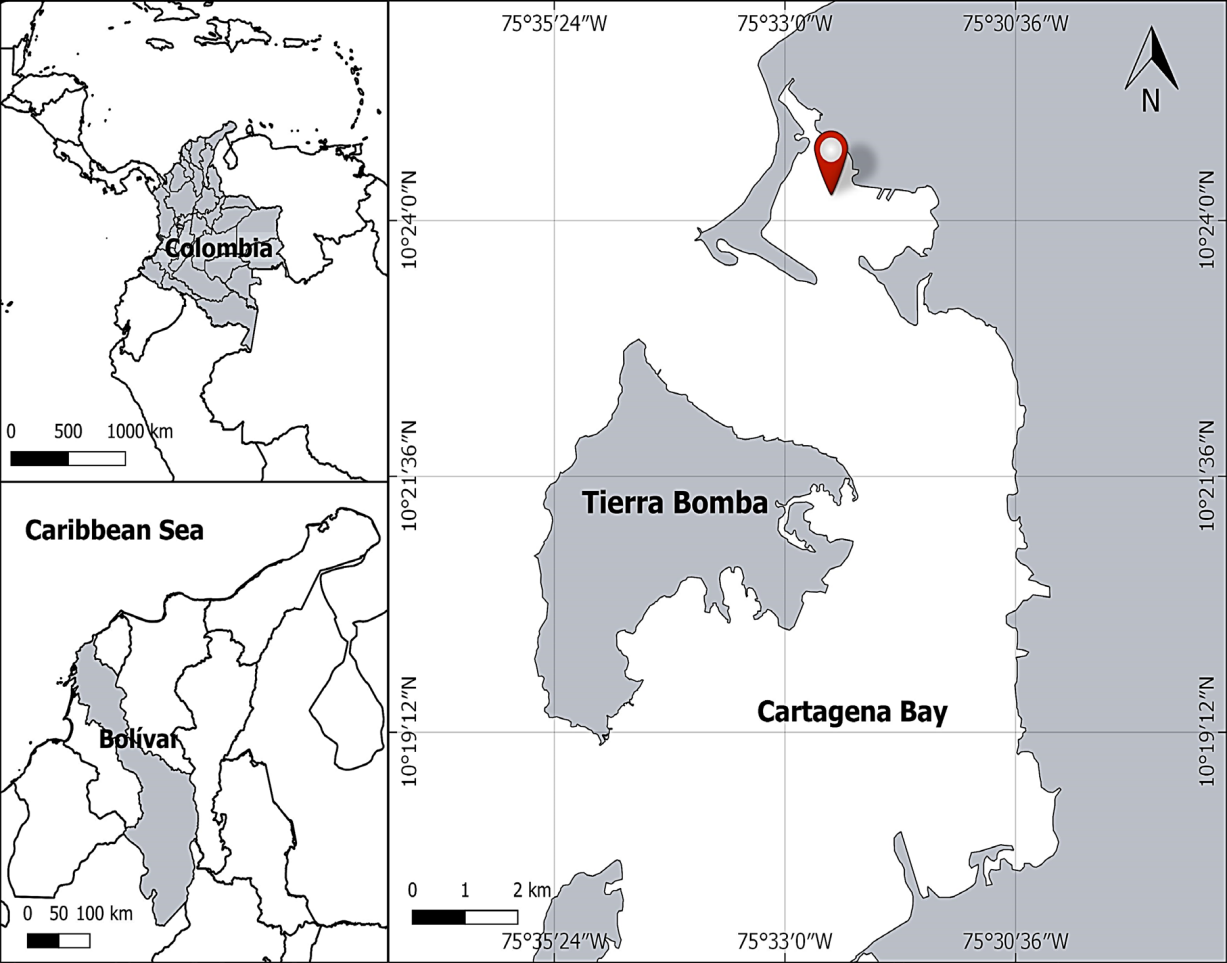
Map of Colombia’s Cartagena Bay indicates the sampling site’s location

Bacterial strains of *Bacillus* spp. BBCOL-009, BBCOL-014 and BBCOL-015 were isolated from sediments of Cartagena Bay (10°25 × 30″N, 75°32 × 25″W), Northern Colombia (Fig. 1). Surface marine sediments were collected 100 m away from the seashore using a Van Veen grab sampler. Once gathered, the material was placed in sterile bags and transported on ice to the microbiology laboratory of the University of Cartagena, for processing.

### Strains isolation and culture conditions

The isolation, purification, and conservation of the bacteria were carried out following the recommendations of Shimkets and Raffie (1990). Fluconazole (0.25 mg/ml) was used to treat pellet samples for three hours prior to inoculation into isolation media. Subsequently, the samples were incubated on NaST21CX agar. Incubating for 15–30 days at 30 °C. Media preparation was performed following the recommendations of Gaspari et al. (2005). Subsequently, individual colonies were taken and spread on Luria–Bertani (LB) agar medium for purification and preservation. Isolated colonies were plated on LB agar until pure cultures were obtained. Three bacterial strains were selected according to variations in the colonies. Later, the colonies were preserved and stored in glycerol (10% w/v) stock at − 80 °C until analysis.

### Molecular identification

#### DNA extraction and PCR amplification of 16 S rDNA

For the DNA extraction, bacterial isolates were grown on LB medium at 30 °C overnight. The cultures underwent centrifugation at 10000 × g for 2 min, and the resulting supernatant was discarded. Genomic DNA extraction was carried out utilizing the QIAamp® DNA Mini Kit (Qiagen, CA, USA) according to the manufacturer’s instructions. To amplify the 16 S rRNA gene, the primer pairs PF (5′-AGAGTTTGATCCTGGCTCAG-3′) and 1492R (5′-ACCTTGTTACGACTT-3′) were used (Wu et al. 2005; Iizuka et al. 2006). The polymerase chain reaction (PCR) was conducted using a Veriti 96-Well Thermal Cycler (Applied Biosystems, Foster City, Ca) thermocycler. The PCR mixtures consisted of 1X AmpliTaq Gold® 360 Master Mix (Applied Biosystems), 0.4 µm of each primer and ~ 100 ng of template in a total reaction volume of 25 µL. The PCR reaction was performed with a hot start of 95 °C for 10 min, followed by 25 cycles of denaturation at 94 °C for 1 min, primer annealing at 43 °C for 1 min and primer extension at 72 °C for 1.5 min, followed by a final extension at 72 °C for 5.5 min. Amplified PCR products of the 16 S ribosomal gene were separated on 1.2% (w/v) agarose gels stained with ethidium bromide (10 mg/ml) and analyzed using a gel documentation system (IngGenius 3 System - Syngene) (Acevedo-Barrios et al. 2019; Bertel-Sevilla et al. 2020).

#### 16 S rDNA sequencing and phylogenetic analysis

PCR products were purified with the QIAquick PCR purification kit (Qiagen, CA, USA), following the standard protocol provided by the manufacturer. Subsequently, automated DNA sequencing was performed by the National Center for Genomic Sequencing-CNSG (Medellin-Colombia) using PF and 1492R primers. The sequence reads obtained were edited and assembled using the CAP3 software (Huang and Madan 1999). The sequence was submitted to GenBank to search for similar sequences with the EzTaxon-e server (http://www.ezbiocloud.net/eztaxon; Kim et al. (2012). Sequence alignments were conducted using the CLUSTAL_W algorithm of MEGA 6 (Tamura et al. 2013), and phylogenetic analysis of all the related 16 S rRNA gene sequences was performed using MEGA 6. Distances were calculated by means of Kimura correction in a pair-wise deletion manner (Kimura 1980). Phylogenetic analyses were inferred using the Neighbour-joining (NJ) (Saitou and Nei 1987), maximum-likelihood (ML) (Felsenstein 1981), minimum-evolution (ME) and maximum-parsimony (MP) (Fitch 1971) methods in MEGA version 6.0 (Tamura et al. 2013). The tree topology was assessed by bootstrap analyses (Felsenstein 1985) based on 1000 resamplings. The sequence of *Lysinibacillus tabacifolii* K3514T with the GenBank accession number JQ754706 was selected as the outgroup to root the phylogenetic tree. The GenBank/EMBL/DDBJ accession numbers for the 16 S rRNA gene sequences of strains BBCOL-009, BBCOL-014 and BBCOL-015 are KU878946, KU878947 and KU878948, respectively.

### Morphology

In order to isolate and describe the morphology of strains, incubation in a VY/2 medium was made, with a pH range of 7 ± 0.2 (agar 1.5%, yeast extract 0.1%, Casitone 0.3%, CaCl_2_·2H_2_O 0.1%,). Growth and morphology were observed with an optical microscope (Olympus BX41). Strains were scraped onto glass slides and stained for identification. The analyzed characteristics of the isolates included colony morphology, staining, spores if any and cell shape. Gram staining was used for microscopic description, according to Koneman et al. (2006) and Breed et al.(1957). To identify the isolated strains, scanning electron microscopy (SEM) was used (Alonso et al. 2009). Growth in LB at several pH (4.0 to 12.0 at 0.5 pH unit intervals) was determined in LB using buffer systems (Zhang et al. 2009).

### Biochemical characterization

Biochemical features were identified using the BBL Crystal™ Kit according to manufacturer instructions (Ashour et al. 2011). Catalase and oxidase activities were detected by bubble production, using hydrogen peroxide solution at 3% (v/v) and the oxidation of Kovac reagent, respectively. Red metile and Voges–Proskauer tests were performed by the conventional battery test according to Winn et al. (2008) and Boone et al. (2005).

### Evaluation of antimicrobial resistance

The Kirby- Bauer disc diffusion method was developed to test the antibiotic sensitivity of the studied strains (Biemer 1973; Murray et al. 1982), using as inhibition substances: ertapenem (E), vancomycine (VA), amoxicillin clavulanate (AMC), penicillin (P), erythromycin (EO) (BBLTM Sensi-DiscTMSusceptibility Test Discs – BD). Briefly, swabs of sterile cotton-tipped were used to transfer the strain to Mueller-Hinton agar plates to produce pure bacterial colonies. Antibiotic discs were put on the plate after the inoculum was dried and subsequently incubated 24–48 h, at 30°. The inhibition of bacterial growth was measured to the nearest millimeter, and their diameters as a measure of the susceptibility of the isolated strain, according to Reller et al. (2009).

### Sodium chloride susceptibility assay

To test hypersaline bacterial growth, the strains were inoculated into LB broth in the presence of NaCl (3.5%, 5.0%, 7.5%, 15% and 30% w/v) by triplicates. The experiments used cell suspension with optical density (OD) = 0.6. The turbidity was recorded after 24 h incubation at 37 °C (Acevedo-Barrios et al. 2016, 2019, 2022, 2023).

### Perchlorate susceptibility assay

In order to identify perchlorate susceptibility, each isolated strain was inoculated in 10 µL of LB broth with concentrations of 250, 500, 750, 1000, 2500, 5000 and 10000 mg/L of perchlorate by triplicates. After 24 h and 37 °C incubation, ClO_4_ˉ concentrations, cell viability and purity of strains were confirmed (Acevedo-Barrios et al. 2016, 2019, 2022, 2023).

### Evaluation of perchlorate reduction by isolates

The experiments used a concentration of 10000 mg/L ClO_4_ˉ in LB medium with 3.5% NaCl, following the inoculation and incubation procedures of susceptibility tests. The final concentration of ClO_4_ˉ was measured with a selective perchlorate electrode (Thermo Fisher) (Acevedo-Barrios et al. 2016, 2019, 2022, 2023).

## Results and discussion

### Molecular Identification

**Fig. 2:**
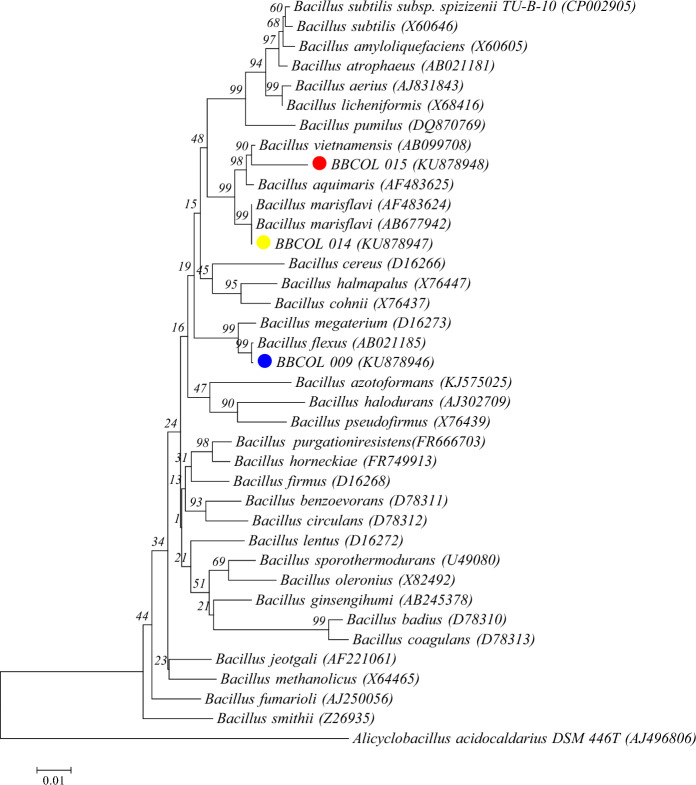
Neighbour-joining tree showing the phylogenetic positions of strains BBCOL-009, BBCOL-014 and BBCOL-015 with other closely related members based on 16 S rRNA gene sequences available from the EMBL database (accession numbers are given in parentheses). The topology of the entire tree was conserved in all trees using different algorithms. The bootstrap values, expressed as percentages at the branching points, indicate the confidence level of the tree branches and were calculated based on 1000 replications. Bar 0.01 nucleotide substitutions per nucleotide position

Regarding the molecular identification of BBCOL-009, BBCOL-014 and BBCOL-015, there were sequenced of 1352, 1360 and 1361 nt of 16 S rDNA, respectively, representing an average of 90% of the *Escherichia coli* 16 S rRNA sequence. The phylogenetic analysis of the 16 S rRNA gene sequence revealed that the isolated bacteria belonged to the genus *Bacillus* spp. within the class Bacilli. The consensus tree illustrating this placement is depicted in Fig. 2. Additionally, the sequence similarities observed with closely related organisms align with the results obtained from the EzTaxon-e server analysis. Almost complete 16 S rRNA gene sequences of three isolated strains were deposited in GenBank (accession numbers: KU878946- KU878948).

EzTaxon-e server search analysis revealed that the strain BBCOL-009 is closely related to *B. flexus* T6186-2 (99.9%, 16 S rRNA gene sequence similarity), *B. paraflexus* RC2(T) (99.2%), *B. megaterium* NBRC 15308 = ATCC 14581(T) (99.1%) and other Bacilli (< 96.6%) respectively. The sequence similarities between strain BBCOL-009 and *B. flexus*, as determined by various clustering algorithms (99% in NJ tree, 99% in ME tree, and 99% in ML tree), in addition to the results from the EzTaxon-e server analysis, consistently indicated that *B. flexus* is the closest relative to strain BBCOL-009.

Strain BBCOL-014 shared highest sequence similarity with *B. marisflavi* TF-11(T), *B. oryzaecorticis* R1(T) and *B. vietnamensis* 15 − 1(T) with 100, 98.7 and 98.4% respectively, and nucleotide differences of 0, 14 and 21 nucleotides respectively. In the phylogenetic tree based on the neighbor-joining algorithm, strain BBCOL-014 joined the cluster comprising *B. marisflavi* with a bootstrap confidence value of 99% (Fig. 2), with which it shares the highest 16 S rRNA gene sequence similarity. The affiliation of strain BBCOL-014 and its closest Neighbour was also supported by the maximum-parsimony and maximum-likelihood algorithms with above 99% bootstrap values. Comparative 16 S rRNA gene sequencing revealed that strain BBCOL-015 was phylogenetically related to *B. vietnamensis* 15 − 1 (T) (98.4% sequence similarity), *B. oryzaecorticis* R1 (T) (98%), *Bacillus aquimaris* TF-12 (T) (98%), and *B. marisflavi* TF-11 (T) (97.5%). Strain BBCOL-015 demonstrated relatively low levels of 16 S rRNA gene sequence similarity compared to other species within the genus *Bacillus*. It formed a clade with *B. vietnamensis*, supported by a bootstrap value of 90% (Fig. 2).

The results revealed that *Bacillus* spp. was the predominant group among the halotolerant bacterial communities in the sediment samples obtained from Cartagena Bay. This finding aligns with previous studies that have identified moderately halophilic bacteria belonging to the genus *Bacillus* in marine environments and related habitats. Examples include *B. marisflavi* and *B. aquimaris* isolated from seawater (Yoon et al. 2003), *Bacillus seohaeanensis* from a solar saltern (Lee et al. 2006), and *B. vietnamensis* from Vietnamese fish sauce (Noguchi et al. 2004).

### Microscopic and biochemical characterization

**Fig. 3:**
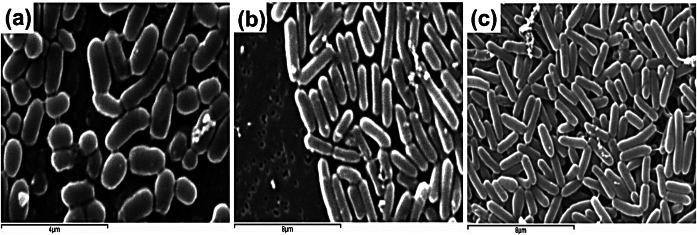
Bacterial isolated from marine sediments. Morphology by SEM. **a** BBCOL-009, **b** BBCOL-014, **c** and BBCOL-015

The morphologic characteristics of the colonies were similar (Fig. 3), presenting a bacterial size average for 1.75 × 0.95 μm, 2.32 × 0.65 μm and 3.08 × 0.70 μm of BBCOL-009, BBCOL-014 and BBCOL-015 respectively. These dimensions are similar to the bacterial *Bacillus* spp. strains described by Priest et al. (1988), Noguchi et al. (2004), and Istock and Graumann (2008). In contrast, Yoon et al. (2003) described a 1500 μm *Bacillus* spp. length. This disparity could be explained by differences in measurement techniques, with scanning electron microscopy (SEM) being one of the most reliable (Reimer 2000).

Strains grew optimally at 37 °C ± 2, pH 7.5 ± 1 and in the absence of NaCl concentrations. All strains had a positive catalase and a negative oxidase activity, except for BBCOL-015, which had a positive oxidase. The three strains metabolized arabinose, sucrose, melibiose, rhamnose, and galactose, while the utilization of mannose, adonitol and inositol was negative. The metabolism of mannitol was positive, except for BBCOL-014. All of the strains had absence of reactions with inositol, sorbitol, indole, ONPG, ornithine, H_2_S production, Voges Proskauer’s, and methyl red. Each strain reduced nitrates. The biochemical characteristics of the strains were 97% compatible with *Bacillus* spp., sustained by the molecular results. The biochemical responses of the three strains are shown in Table 1.

**Table 1:** Comparison of morphological and biochemical characteristics of isolated strains BBCOL-009–BBCOL-014, and BBCOL-015 with *B. flexus* DSM1320, *B. marisflavi* TF-11, and *B. vietnamensis* sp. nov

Characteristic	BBCOL-009	BBCOL-014	BBCOL-015	DSM1320*	TF-11^ǂ^	*B. vietnamensis *sp. nov °
Color of colony	Yellow	Yellow	yellow	NR	Pale Yellow	NR
Morphology	Rod shaped	Rod shaped	Rod shaped	Rod Shaped	Slightly irregular	Rod Shaped
Length (µm)	1.75 μm	2.32 μm	3.08 μm	NR	1000–2000 μm	2.0–3.0 μm
Thickness (µm)	0.95 μm	0.65 μm	0.70 μm	NR	NR	0.5–1.0 μm
Motility	−	−	−	−	+	+
Gram straining	+	+	+	+	+/V	+
Endospore	+	+	+	+	+	+
Spore position	Central	Central	Subterminal	Central	Central/Subtermial	Central
Oxidase	−	−	+	−	−	+
Catalase	+	+	+	+	+	+
Arabinose	+	+	+	NR	+	−
Mannose	−	−	−	+	+	−
Sucrose	+	+	+	+	+	NR
Melibiose	+	+	+	+	+	NR
Rhamnose	+	+	+	+	+	NR
Mannitol	+	−	+	+	+	+
Adonitol	−	−	−	NR	−	NR
Galactose	+	+	W	NR	W	−
Inositol	−	−	−	NR	NR	NR
p-n-p-phosphate	−	−	+	NR	NR	−
p-n-p a-ß-glucoside	V	−	−	NR	+	−
p-n-p-ß-galactoside	+	+	+	NR	+	−
Prolinenitroanilide	−	−	−	NR	+	NR
p-n-p bis-phosphate	−	−	−	NR	NR	NR
p-n-p-xyiloside	+	+	+	NR	+	−
p-n-p-a-arabinoside	+	+	+	NR	+	−
p-n-p-phosphorylcholine	−	−	−	NR	NR	NR
p-n-p-ß-glucuronide	−	−	−	NR	NR	NR
p-n-p-N-acetylglucosamide	−	−	−	NR	NR	NR
γ-L-glutamyl p-nitroanilide	−	−	−	NR	NR	NR
Aesculin	+	+	+	NR	+	+
p-nitro-DL-phenylalanine	−	−	−	−	NR	NR
Urea	−	−	−	−	NR	−
Glycine	−	−	−	NR	NR	NR
Citrate	V	−	W	+	NR	NR
Malonicacid	+	+	+	NR	NR	NR
Triphenyltetrazoliumchloride	+	+	+	NR	NR	NR
Lactose	+	+	+	+	+	NR
Bacteriolytic Capacity	+	+	+	NR	+	+
Cellulolytic Capacity	−	−	−	−	NR	NR
Nitrate Reduction	+	+	+	NR	NR	+
Indole	−	−	+	−	NR	+
ONPG	−	−	−	NR	NR	NR
Ornitine Utilization	−	−	−	NR	NR	NR
H_2_S Production	−	−	−	NR	NR	NR
Voges Proskauer’s	−	−	−	−	NR	NR
Methyl red	−	−	−	−	NR	NR
Sorbitol	−	−	−	NR	NR	NR
Hemolysis	α	α	α	NR	NR	NR

+, Positive reaction; − negative reaction; W, weakly positive reaction; V, variable reaction; NR, not reported; H, Halfway. * *B. flexus* DSM1320 (Priest et al. 1988) ǂ *B. marisflavi* TF-11 (Yoon et al. 2003). °*B. vietnamensis* sp. nov (Noguchi et al. 2004)

### Antimicrobial resistance test

The responses of the three strains to the antimicrobial test substances are shown in Table 2. All the evaluated strains presented resistance to Erythromycin. This finding suggests the presence of the *erm* gene (erythromycin ribosome methylation), which grants the strain the ability to produce methylase, which modifies their 23 S sRNA, as well as *mef* (provides resistance to 14 and 15 carbon macrolides by expulsion pump) (Davies and Davies 2010). However, this resistance has been reported mainly in grampositive cocci (Yang et al. 2024; Weisblum 1995), nonetheless, genes are acquired through the transference of transposons, probably by genetically modified microorganisms in the environment, previously exposed to antibiotics.

**Table 2:** Antibiotic sensitivity of isolated strains BBCOL-009−BBCOL-014, and BBCOL-015

Inhibition substances	BBCOL-009	BBCOL-014	BBCOL-015	DSM1320*	TF-11^ǂ^	B. vietnamensis sp. nov °
Amoxicillin clavulanate	(R)	1.4 cm(S)	1.1 cm (S)	NR	NR	NR
Vancomycine	(R)	1.1 cm(S)	1.3 cm (S)	NR	NR	NR
Penicillin	(R)	1.0 cm(S)	1.0 cm (S)	NR	NR	NR
Ertapenem	(R)	(R)	(R)	NR	NR	NR
Erythromycin	(R)	1.9 cm(S)	1.5 cm (S)	NR	NR	NR

R, resistant; S, Sensible. * *B. flexus* DSM1320 (Priest et al. 1988). ǂ *B. marisflavi* TF-11 (Yoon et al. 2003). °*B. vietnamensis* sp. nov (Noguchi et al. 2004)

In addition, the strains revealed resistance to amoxicillin clavunate, vancomycine, penicillin, and ertapenem (Table 1). This behavior has been reported by Zhang et al. (2014), indicating the high capacity to activate metabolic pathways that enable the bacteria to survive in hostile conditions.

The fact that our strains were capable of resisting the aforementioned molecules indicates the property of this microorganisms to degrade pharmaceuticals and personal care products (PPCPs), considered as concerning emerging pollutants, negatively affecting aquatic ecosystems since there are no clear analyses that regulate or quantify the concentrations allowed in these environments, as well as their impact on the microbiota, flora, and fauna, is not clearly established (Liu and Wong 2013), therefore, they can be considered as potential bio-inputs in the area of biotechnology and bacterial bioremediation of emerging pollutants (e.g. other drugs like analgesics, β-blockers, preservatives, UV Filters, among others), due to structural homology. Although further studies must be done to prove the potential degradation capacity of the studied strains, the results shown in this study show the first step to consider choosing the bacterial strains, as an input for biotechnology.

###  Evaluation of tolerance to perchlorate, NaCl, and pH variations

This study showed the capacity of the isolated strains BBCOL-009, BBCOL-014 and BBCOL-015 to grow at concentrations of 7.5% w/v and above of NaCl (Table 3), confirming them as moderate halotolerant species (Albuquerque et al. 2008; Zhang et al. 2009; Lei et al. 2014; Bahamdain et al. 2015). In addition to NaCl, these strains were capable to grow and tolerate KClO_4_^−^ from 250 to 10000 mg/L. The NaCl and KClO_4_^−^ tolerance and degrading capacity of the strains were demonstrated to be related to biofilm formation, which was present in the studied cultures. This biofilm is a physical barrier composed of extracellular polymeric substances and biomass, indicating the strains’ ability to isolate themselves from the media after being stressed by high salt concentrations via a concentration gradient (Souid et al. 2021).

**Table 3:** Growth and development of BBCOL-009, BBCOL-014 and BBCOL-015 in presence of NaCl and KClO_4_^−^ and survival within pH changes

Conditions	Concentrations	BBCOL-009	BBCOL-014	BBCOL-015
NaCl (% w/v)	3.5% *^✝^	R	R	R
	5.0% *^✝^	R	R	R
	7.5% *^✝^	R	R	R
	15%*^✝^	S	S	S
	30% *^✝^	S	S	S
KClO_4_^-^ (mg/L)	250 *^✝^	R	R	R
	500 *^✝^	R	R	R
	750 *^✝^	R	R	R
	1000 *^✝^	R	R	R
	2500 *^!^	R	R	R!
	5000 *^!^	R	R	R!
	10000 *^!^	R	R	R!
pH	7.0 ± 0.5*^!^	+	+	+
	6.5 to 10.0*	+	+	+

* Growth at optimal temperature (37 °C ± 0.5). *Growth at optimal pH (7.0 ± 0.5). ! Biofilm formation. S, Sensible R, Resistant. + Bacterial growth

Regarding pH as a condition for growth and development of the studied strains, the isolated strains showed a high capacity to resist alkaline concentrations (8 to 12 ± 0.5). This behavior suggests that *Bacillus* spp. are facultative alkalophilic microorganisms. Several studies conducted by Sanchez-Gonzalez et al. (2011), agree that *Bacillus* spp. are capable of growing and developing under alkaline conditions (Sanchez-Gonzalez et al. 2011). Due to their alkaline-resistance attribute, it is important to detect and conserve these alkalophilic microorganisms (Mandal et al. 2013; Roy and Mukherjee 2013) for their potential production of alkaline active enzymes for the treatment of alkaline sewage derivatives from the laundry detergent industry (Roy and Mukherjee 2013; Coelho et al. 2016; Lucena-Padrós and Ruiz-Barba 2016﻿).

### Evaluation of perchlorate reduction by isolates

**Fig. 4:**
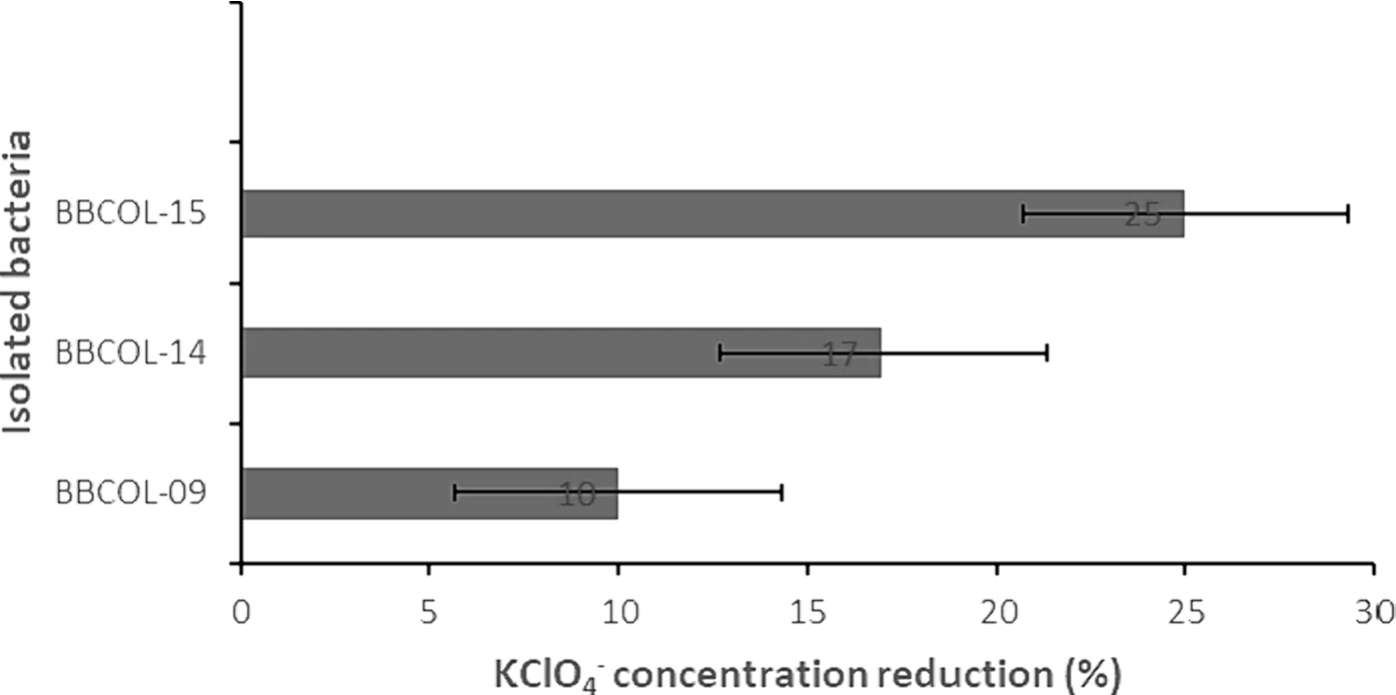
Percentage of KClO_4_^−^ reduction of the bacteria BBCOL-009, BBCOL-014 and BBCOL-015 from saline environments in the sediments in Cartagena Bay. Effect of the 24 h contact time, optical density at OD 600, and optimal pH (7.0)

In this study, the bacterial strains BBCOL-009, BBCOL-014 and BBCOL-015 reduced perchlorate concentrations by 10, 17 and 25%, respectively (Fig. 4). Table 4 illustrates the variety of perchlorate-reducing species.

The bacterial strains BBCOL-009, BBCOL-014, and BBCOL-015 reduced perchlorate close the values of perchlorate reduction reported for *Bacillus* isolated from marine sediments of the Colombian Caribbean and Antarctica (Acevedo-Barrios et al. 2019, 2022) (Table 4). The Betaproteobacteria class is the most commonly detected perchlorate-reducing bacteria. nonetheless, BBCOL-009, BBCOL-014 and BBCOL-015, of this study, offer a promising resource for the bioremediation of perchlorate-polluted environments and matrix.

**Table 4:** Genus and species of perchlorate-reducing bacteria

Bacterial genus and species	Percentage of reduction (%)	Environmental conditions	isolation site	Reference
*Nesiotobacter* sp	25	FC, 37 °C	Saline soils from Caribbean coast, Colombia	Acevedo-Barrios et al. 2019; Acevedo-Barrios and Olivero-Verbel 2021)
*Bacillus vallimostis*	23			
*Salinivibrio costicola*	25			
*Vibrio* sp.	14			
*Bacillus* sp.	12			
*Staphylococcus* spp.	10			
*Psychrobacter cryohalolentis*	30.3	FC, 4 °C	Soil, Deception Island, Antarctica	(Acevedo-Barrios et al. 2022)
*Psychrobacter urativorans*	32.6			
*Psychrobacter nivimaris*	22		Soil, Half Moon Island, Antarctica	
*Sporosarcina aquimarina*	21.8			
*Pseudomonas lactis*	21.6			
*Idiomarina loihiensis*	40		Soil, Horseshoe Island, Antarctica	
*Bacillus* sp.	19			
*Rhodococcus sp.*	45	Anaerobic	Sludge from municipal Wastewater treatment plant, South Korea	(Lee et al. 2015)
*Dechloromonas sp.*	NR	FC	groundwater and soil, Army ammunition plant, USA	(Shrout et al. 2005)
*Desulfomicrobium sp.; Thauera sp.* consortia	100	Anaerobic	Wastewater plant, China	(Wan et al. 2016)
*Azospira sp.*	100	FC	wastewater treatment plant in Suwon, Korea.	(Nam et al. 2016)
*Magnetospirillum sp.*	NR	FC	Lake sediments, India	(Jacob et al. 2018)
*Haloterrigena sp.*	NR	FC	Atacama Desert, Chile	(Flores et al. 2020)
*Pseudomonas stutzeri*	94	Aerobic	Soils from industrial area, India	(Shete et al. 2008)
*Arthrobacter sp.*	92			
*Wolinella succinogenes, Dechloromonas agitata* consortia	31−100	FC	Saline lake sediment, China	(Dong et al. 2022)

*FC* Facultative conditions, *NR* Not reported

A variety of perchlorate-reducing bacterial species can reduce this contaminant; however, the percentage of reduction varies according to genus and the period of exposure to the pollutant. The rates of perchlorate reduction determined in this study were comparable to those reported by Acevedo-Barrios et al. (2019), Acevedo-Barrios et al. (2022), Acevedo-Barrios et al. (2023).

Most perchlorate-reducing bacteria are anaerobic and facultative, and molecular oxygen is produced as an intermediate for microbial perchlorate reduction in a process that exudes nitrate (Acevedo-Barrios et al. 2022) and use this contaminant as an electron acceptor in their metabolic reactions. Microbial reduction of ClO_4_^−^ occurs via the biochemical reaction is ClO_4_^−^ → ClO_3_^−^ → ClO_2_^−^ → Cl^−^ +O_2_). Enzymes, such as perchlorate reductase and superoxide chlorite, carry out the reduction or elimination of perchlorate (Acevedo-Barrios et al. 2019, 2022; Acevedo-Barrios and Olivero-Verbel 2021; Acevedo-Barrios et al. 2023). A reductase can reduce perchlorate to chlorate, and subsequently, to chlorite, whereas superoxide chlorite changes chlorite to chloride and molecular oxygen (Acevedo-Barrios et al. 2019). Biological reduction of perchlorate using bacteria completely degrades perchlorate ions into Cl^-^ and O_2_ (Acevedo-Barrios and Olivero-Verbel 2021).

### Application potential in perchlorate remediation and astrobiology

This study showed for the first time the high capacity of Colombian Caribbean Coast bacterial isolates to survive in extreme environments as well as their capacity to reduce perchlorate. These strains are potentially inputs for biotechnological, industrial, and medical processes, including the biodegradation of several xenobiotics. Lundberg et al. (2013), demonstrated the use of *Bacillus* spp. as an enzyme producer. In addition, the use of *Bacillus* spp. was reported to be a biofuel producer and biodegradator of biodiesel and other pollutants (Lundberg et al. 2013; Mukhtar et al. 2019; Acevedo-Barrios et al. 2019; Bertel-Sevilla et al. 2020). Moreover, Mukhtar et al. (2019) related the presence of halotolerant bacterial strains with lipases and hemicellulose degradation capacity, indicating the importance of these microorganisms in soil fertilization and plant metabolism.

In the context of sustainability, the utilization of bacterial-mediated processes to address perchlorate contamination is gaining interest. These bacteria have evolved mechanisms to degrade perchlorate, offering a biological potential for remediation, however, it is imperative to understand their applicability and limitations as well as the diversity of available technologies, ranging from in situ ex situ approaches and natural attenuation to controlled bioreactors at different scales (Fang and Naidu 2023). Table 5 provides a list of perchlorate bioremediation technologies.

**Table 5:** Technologies for perchlorate bioremediation

Technology/ Bioreactor type	Treatment phase	Fundamentals	Operation conditions	References
Permeable Reactive Barriers (PRBs)	Liquid	Barriers packed with materials promoting growth of perchlorate-reducing bacteria.	Anaerobic. Electron donors like organic substrates. Neutral pH preferred.	(Borden 2007)
Fluidized Bed Reactors (FBR)	Liquid	Liquid phase with fluidized media supporting biofilm growth.	Anaerobic. Electron donors like ethanol. 20 to 37 °C, neutral pH.	(Hatzinger et al. 2002)
Sequencing Batch Reactors (SBR)	Liquid	Batches treated in series of steps in one tank.	Anaerobic. Electron donors like acetate or ethanol. 20 to 30 °C, neutral pH.	(Nor et al. 2011; Kim et al. 2020)
Biofilm Reactors	Liquid	Continuous-flow system with surfaces promoting growth of biofilms for treatment.	Anaerobic. Electron donors like methane, acetate, hydrogen gas. 15 to 35 °C, neutral to slightly alkaline pH.	(Xie et al. 2018)
Packed Bed Reactors (PBR)	Liquid	Columns with high surface medium supporting bacterial growth.	Anaerobic. Electron donors like acetate. 20 to 35 °C, pH ~ 7.	(Losi et al. 2002)
Membrane bioreactor (MBR)	Liquid	activated sludge and solid–liquid separation using microfiltration	Electron donor tiosulfate,30 °C, pH ~ 7.5.	(Yilmaz et al. 2023)
Biostimulation	Solid	Adding nutrients to soil to stimulate indigenous bacteria. Ex-Situ or In-Situ.	Anaerobic. Electron donors added like acetate. Local soil temperature and pH.	(Nozawa-Inoue et al. 2005)
Bioaugmentation	Solid	Adding inoculum and nutrients to soil. Ex-Situ or In-Situ.	Anaerobic or aerobic. Electron donors added like acetate. Local soil temperature and pH.	(Shete et al. 2008; Shang et al. 2018)
Natural attenuation	Solid	Monitored transformation by indigenous microorganisms. In-Situ.	Aerobic-Anaerobic. Local soil temperature and pH, variable water saturation and aeration conditions.	(Gal et al. 2008)

The performance of strains BBCOL-009, BBCOL-014, and BBCOL-015 in perchlorate reduction across a wide pH range and saline conditions highlights their suitability for various remediation techniques. These bacteria exhibit resistance to saline conditions and adaptability to different pH levels (Ma et al. 2023; Adams et al. 2015). Their adaptability to broad pH ranges makes them valuable inputs for bioreactors and in situ processes, mitigating potential challenges associated with less adaptable strains (Ma et al. 2023).

Physicochemical properties, especially pH, profoundly impact bacterial growth, metabolism, and survival. Understanding the complex relationship between pH and bacterial performance is crucial in microbiology, environmental sciences, and clinical research (Saravanan et al. 2023). Bacteria capable of resisting extreme pH levels play vital roles in acidic or alkaline ecological niches, thus expanding knowledge about such environments (Harrison et al. 2013).

Operational and pilot-scale variables must be tested, as each technology, aimed at specific environmental matrices, presents inherent advantages and challenges. For example, electron donors are crucial in the perchlorate reduction process. Acetate is the most common, but the role of hydrogen, ethanol, or lactate, as well as the initial perchlorate concentration and electron donor ratio, has been investigated (Losi et al. 2002; Xie et al. 2018; Kim et al. 2020; Nozawa-Inoue et al. 2005; Gal et al. 2008; Shete et al. 2008; Nam et al. 2016).

Environmental risks, including antibiotic resistance, must be evaluated during technological scalability. While initial antibiotic resistance suggests sustained efficiency in perchlorate reduction, prolonged exposure may lead to antimicrobial resistance gene expression and microbiota alterations (Zheng et al. 2019).

The study of such extremophilic microorganisms is not limited to bioremediation; it also offers valuable insights for astrobiology, especially in Mars exploration, where *Bacillus* spp. could assist in perchlorate reduction, potentially addressing challenges in food production during manned missions (Oze et al. 2021; Schuerger et al. 2003; Nicholson et al. 2012).

Future studies exploring the astrobiological potential of Cartagena sectors, characterized by sandstone formations, hold promise, considering the presence of similar formations and perchlorates on Mars (Yen et al. 2017; Schieber et al. 2020; Farley et al. 2016; Martin et al. 2020). This study marks a fundamental step towards understanding extremophilic microorganisms in the region and their astrobiological and bioremediation potential.

These bacteria could be advantageous adaptive, with a broad temperature tolerance and a pH range (Ma et al. 2023; Adams et al. 2015). Our strains showed strong adaptiveness to a wide range of pH, which demonstrated them to be a highly adaptative input for using in bioreactors and in situ processes, due to potential of hydrogen could be a tedious task in less adaptative strains, decreasing efficiency. This behavior has been reported in other bacterial strains such as *Exiguobacterales, Bacillales, Lactobacillales* and *Bacillales* (Ma et al. 2023), showing, similarly to ours, biofilm formation.

Among the physicochemical properties related to bacterial performance of growth, metabolism, and survival, the pH is one of the most important. The pH of the surrounding medium can influence the solubility of essential nutrients, the efficiency of metabolic pathways, and the effectiveness of antimicrobial agents. Understanding the intricate relationship between pH and bacterial performance is essential in various fields, including microbiology, environmental science, and clinical research (Saravanan et al. 2023). The impact of pH on bacterial performance is particularly significant in extreme environments, and understanding the adaptations of bacterial strains that resist extreme pH levels is crucial, especially in the context of astrobiology. Bacteria that exhibit tolerance to extreme pH conditions play a pivotal role in ecological niches with acidic or alkaline features, such as acid mine drainage sites, alkaline soda lakes, or geothermal springs. These extremophiles have evolved unique mechanisms to thrive in environments outside the typical pH range. Studying extremophiles not only expands our knowledge of microbial diversity but also has implications for astrobiology, where the exploration of extraterrestrial environments involves considerations of extreme pH conditions. The ability of certain bacterial strains to withstand extreme pH levels is relevant to the search for life beyond Earth, as extraterrestrial environments may present challenges akin to those encountered by extremophiles on Earth. Therefore, investigations into the pH tolerance of bacterial strains hold significance not only for understanding microbial ecology on Earth but also for anticipating and exploring potential habitats in the broader scope of astro biological research (Harrison et al. 2013).

The ability to generate biofilms and adherence to surfaces made them suitable, especially for reactor-based systems or solid-phase treatments. It is a fact that in the mass production of Polydroxialkanoate (PHA), common in biofilm formation in *Bacillus* genus, BBCOL-009 stands out among the rest of the PHA-producers due to its capacity to tolerate extreme conditions that could be present within the bioreactor (Divyashree et al. 2009). Highlights that biofilms are shared spaces in a bacterial population.

However, pilot scale and operational variables should be tested as each technology, targeted for specific environmental matrices, comes with inherent advantages and challenges. For instance, electron donors are crucial in the perchlorate reduction process. The most common is acetate, however, the role of hydrogen, ethanol, or lactate, organic matter has been investigated (Losi et al. 2002; Xie et al. 2018; Kim et al. 2020). Also, the efficiency of reduction depends on the initial perchlorate concentration and the perchlorate electron donor ratio (Nozawa-Inoue et al. 2005; Gal et al. 2008; Shete et al. 2008) and the negative effect of nitrate has been reported (Nam et al. 2016).

Few environmental risks must be considered in the scaling of remediation technologies. An important aspect is antibiotic resistance, in which the strains showed resistance (Table 2). This condition initially suggests the capacity of the strains to sustain their efficiency in perchlorate reduction in environments containing environmental pollutants such as antibiotic’s traces, which are considered emerging pollutants. However, prolonged exposure may generate the expression of antimicrobial resistance genes and alterations in the microbiota. This should be investigated in depth (Zheng et al. 2019). The remediation species should maintain genetic stability, ensuring consistent perchlorate reduction and environmental safety over time.

All the above indicate that these microorganisms offer an opportunity to delve into other fields of study, such as astrobiology. One of the main lines of research in this transdisciplinary field (Leal et al. 2023) is the study of extremophile microorganisms that can serve as a model for understanding possible biological adaptations in other bodies of the Solar System (DasSarma et al. 2020; Thombre et al. 2020). Some studies have shown that organisms of the genus *Bacillus* could be promising in searching for potential past or present life on Mars, mainly due to the presence of resistance structures such as endospores (Schuerger et al. 2003; Nicholson et al. 2012).

In addition, the results provide a much more interesting perspective, such as the ability of *Bacillus* spp. to grow in environments contaminated with perchlorate and allow the reduction of this salt in the substrate. This is a fundamental aspect in astrobiology studies on Mars since perchlorate has been detected on the surface of the red planet, being one of the main problems for food production in future manned missions (Oze et al. 2021). However, organisms such as those reported in the present study could be a potential solution to this problem.

Although no study was carried out on the geological origin of the sediments sampled and analyzed in the present study, a future perspective to continue delving into the astrobiological potential of some Cartagena sectors is based on fine to very fine-grained sandstones’ characterizing their geological formations. Slightly feldspathic, with few lithics and parallel flat lamination in thin layers (Clavijo and Royero 2000). They are presenting the region as a potential study site of astrobiological interest, taking into account that the presence of sandstones has been identified in the Gale Crater on Mars (Yen et al. 2017; Schieber et al. 2020), as well as the presence of perchlorates (Farley et al. 2016; Martin et al. 2020). Thus, this study is presented as a first step in a chain of opportunities to continue delving deeper into the study of the extremophile microorganisms in the region and their astrobiological and bioremediation potential.

## Conclusion

This is the novel study conducted in Bahia Cartagena, Colombian Caribbean, which reveals that bacteria belonging to the genus *Bacillus*, specifically BBCOL-009, BBCOL-014, and BBCOL-015, were extracted from marine sediment samples obtained from Cartagena Bay. It was found that these bacteria exhibit tolerance to environmental perchlorate concentrations of up to 10000 mg/L. Moreover, they are capable of reducing perchlorate concentrations by 10–25%. Furthermore, these bacteria demonstrate the ability to thrive in a wide range of pH and NaCl concentrations, showcasing their potential for remediating this contaminant through various technologies and biotechnological applications in agriculture and water sources remediation. Although the primary focus of the study of perchlorate-reducing and resistant bacteria is in the ecological and agricultural realms, from an astrobiological perspective, perchlorate-resistant bacteria serve as models for astrobiological investigations.

## Data Availability

No datasets were generated or analysed during the current study.
